# A Digital Cognitive Aid for Anesthesia to Support Intraoperative Crisis Management: Results of the User-Centered Design Process

**DOI:** 10.2196/13226

**Published:** 2019-04-29

**Authors:** Stefanie Schild, Brita Sedlmayr, Ann-Kathrin Schumacher, Martin Sedlmayr, Hans-Ulrich Prokosch, Michael St.Pierre, German Cognitive Aid Working Group

**Affiliations:** 1 Department of Medical Informatics, Biometrics and Epidemiology Chair of Medical Informatics Friedrich-Alexander University Erlangen-Nürnberg Erlangen Germany; 2 Center for Evidence-based Healthcare Carl Gustav Carus University Hospital Technische Universität Dresden Dresden Germany; 3 Carl Gustav Carus Faculty of Medicine Institute for Medical Informatics and Biometry Technische Universität Dresden Dresden Germany; 4 Anästhesiologische Klinik Universitätsklinikum Erlangen Erlangen Germany; 5 Berufsverband Deutscher Anästhesisten Nürnberg Germany

**Keywords:** anesthesiology, checklist, crew resource management, healthcare, emergency treatment, ergonomics, human factors, practice guideline, reference books, medical, resuscitation, user-computer interface

## Abstract

**Background:**

Stressful situations during intraoperative emergencies have negative impact on human cognitive functions. Consequently, task performance may decrease and patient safety may be compromised. Cognitive aids can counteract these effects and support anesthesiologists in their crisis management. The Professional Association of German Anesthesiologists set up a project to develop a comprehensive set of digital cognitive aids for intraoperative emergencies. A parallel development for several software platforms and stationary and mobile devices will accommodate the inhomogeneity of the information technology infrastructure within German anesthesia departments.

**Objective:**

This paper aimed to provide a detailed overview of how the task of developing a digital cognitive aid for intraoperative crisis management in anesthesia was addressed that meets user requirements and is highly user-friendly.

**Methods:**

A user-centered design (UCD) process was conducted to identify, specify, and supplement the requirements for a digital cognitive aid. The study covered 4 aspects: analysis of the context of use, specification of user requirements, development of design solutions, and evaluation of design solutions. Three prototypes were developed and evaluated by end users of the application. Following each evaluation, the new requirements were prioritized and used for redesign. For the first and third prototype, the System Usability Scale (SUS) score was determined. The second prototype was evaluated with an extensive Web-based questionnaire. The evaluation of the third prototype included a think-aloud protocol.

**Results:**

The chosen methods enabled a comprehensive collection of requirements and helped to improve the design of the application. The first prototype achieved an average SUS score of 74 (SD 12), indicating good usability. The second prototype included the following main revisions: 2-column layout, initial selection of patient type (infant, adult, or parturient), 4 offered search options, and the option to check off completed action steps. Its evaluation identified the following major revision points: add quick selection for resuscitation checklists, design the top bar and tabs slightly larger, and add more pictograms to the text. The third prototype achieved an average SUS score of 77 (SD 15). The evaluation of the think-aloud protocol revealed a good intuitiveness of the application and identified a missing home button as the main issue.

**Conclusions:**

Anesthesiology—as an acute medical field—is particularly characterized by its high demands on decision making and action in dynamic, or time-critical situations. The integration of usability aspects is essential for everyday and emergency suitability. The UCD process allowed us to develop a prototypical digital cognitive aid, exhibiting high usability and user satisfaction in the demanding environment of anesthesiological emergencies. Both aspects are essential to increase the acceptance of the application in later stages. The study approach, combining different methods for determining user requirements, may be useful for other implementation projects in a highly demanding environment.

## Introduction

### Background

Intraoperative emergencies require rapid, coordinated management in time-critical and stressful settings. Anesthesiologists are faced with the twofold expectation that their situational assessment should include all possible differential diagnoses, whereas their management should always be grounded in current evidence-based recommendations. Unfortunately, stressful situations have a negative impact on human cognitive functions (eg, attention, working memory, or prospective memory) and analysis-driven decision making [1]. Challenged by an uncommon emergency, task performance can decrease even more, as humans are not optimized to retrieve rarely used information. As a result, omissions of critical steps, practice variability, and noncompliance to established guidelines increase [2].

Encouraged by promising results from other high-profile industries in which very complex problem situations have to be solved quickly, the past decade has witnessed a growing interest of anesthesiologists in paper-based or electronic cognitive aids that can counteract the possible deleterious effects of stress listed above [3,4]. Cognitive aids can remind clinicians of important diagnostic causes, guide them on the basis of current evidence-based practices through a sequence of complex steps, and prevent them from omitting key actions [5]. Furthermore, these aids should anticipate common pitfalls of the particular emergency, provide prioritized and explicit instructions to prevent them, and contain important local information (eg, phone numbers, depositories of critical drugs, and precalculated drug dosages) that may help increase the speed and fluidity of performance. A process of user-centered design (UCD) is necessary to prevent poorly designed aids from distracting and negatively affecting clinicians. Ideally, a national body should attempt, in a formal consensus approach, to create a more comprehensive set of perioperative cognitive aids than what is currently available [6].

### Objectives

As there are currently no officially endorsed cognitive aids for intraoperative emergencies available in Germany, the Professional Association of German Anesthesiologists (BDA) set up a project to develop such a comprehensive set of digital cognitive aids for intraoperative emergencies. Within the scope of this study, clinicians from different university hospitals (German Cognitive Aid Working Group) worked together with human factors engineers and software developers. To ensure wide dissemination of the cognitive aid to German anesthesia departments, consideration had to be given to the inhomogeneity of information technology infrastructures within German anesthesia departments. As a result, it was decided to develop an electronic cognitive aid as a mobile health app for multiple software platforms as well as stationary and mobile devices.

In this paper, we have answered the following research questions:

1. What demands do clinicians have on the digital representation of cognitive aids for emergency situations?
2. How can we best develop a user interface for a cognitive aid to be applied in the time-critical and stressful environment of intraoperative crisis management in anesthesia?

## Methods

### User-Centered Design

The development of this cognitive aid is based on the UCD process of the International Organization for Standardization (ISO) norm 9241-210 [7], which has recently been performed for the development of other applications in the medical context [8-10]. This process comprises the analysis of the context of use, the definition of user requirements, the development of prototypes, and tests with representative users.

For this study, a thorough analysis of the physical and organizational environment, the application context, and the technical and task-specific requirements of the end users was performed. These requirements were implemented in an initial prototype and evaluated with a user test. The evaluation was used to query the user requirements regarding their prioritization and, if necessary, to adapt them accordingly or add new derived ones. With the user requirements revised in this way, a second prototype was created, which in turn was evaluated with a user test. This iteration step was repeated with a third prototype.

**Figure 1:**
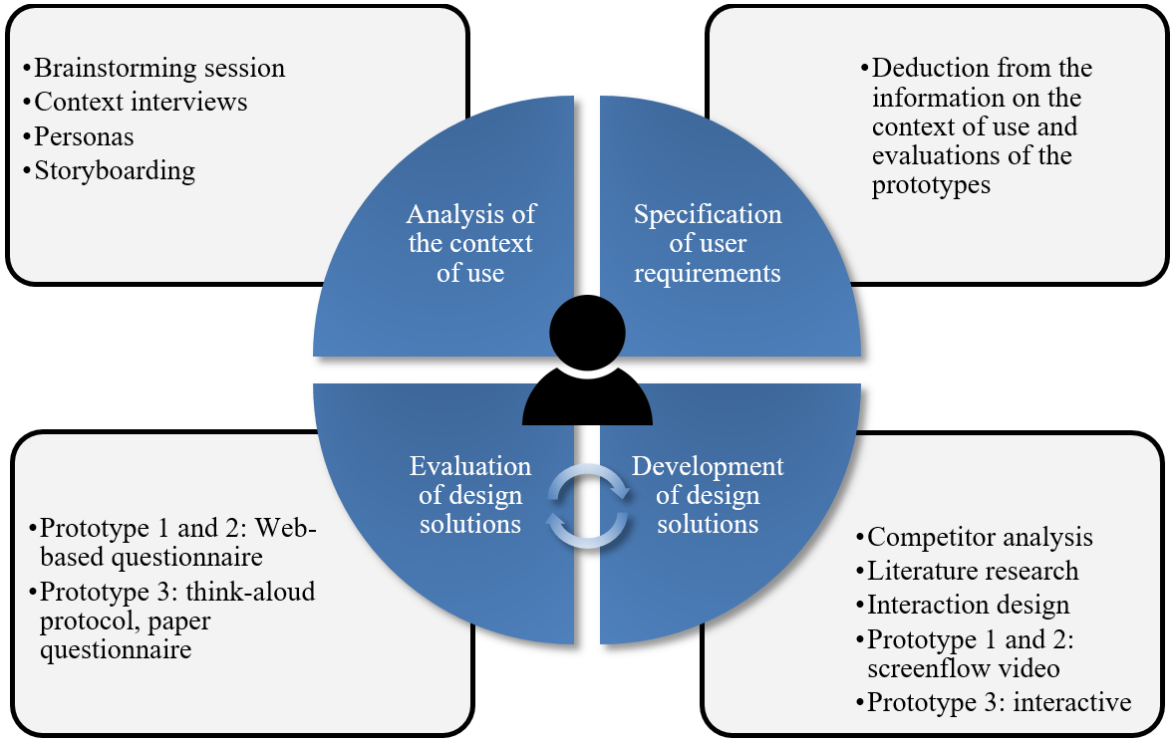
Study process design.

The study process design [7] is presented in Figure 1. Owing to its scope, only the more significant parts of the process are described in this paper.

### Analysis of the Context of Use

A brainstorming session was held with 7 members of the German Cognitive Aid Working Group present at an initial meeting to identify users’ expectations regarding a digital cognitive aid and develop a first set of ideas concerning its functionality. Furthermore, context interviews with 4 anesthesiologists and 3 anesthetic nurses were conducted at the anesthesia departments of 3 project partners (University Hospital Erlangen, University Hospital Dresden, and Trauma Hospital Berlin). Personas, that is, written representations of the intended end users of the application, were developed to give a clear picture of the characteristics of the users within the project group.

On the basis of the results of the above methods, storyboards were developed for 3 different use cases (intraoperative management of malignant hyperthermia, management of ventricular fibrillation, and a local user editing the content). To visualize the respective stories, the interaction between users and the application was illustrated by images and accompanying text. The 3 storyboards were shared with all members of the project group to get general feedback on the stories. They were asked to indicate how well the application met their needs to derive additional requirements for the application. Feedback was given in free-text form and aggregated afterward. Subsequently, the storyboards were slightly modified by the additional requirements identified.

### Specification of User Requirements

The collected information by the various applied methods was consolidated and recorded in individual documents. The main requirements document contained the results of the user context analysis and evaluation of the first prototype together with the specification of the respective derived and prioritized user requirements. In addition, 2 further documents included the evaluation results of the second and third prototype and the respective derived or revised requirements.

If the evaluation result of a prototype regarding the need for revision was ambiguous in some aspects, relevant issues were identified and prepared both textually and visually. Subsequently, the points of discussion were jointly reviewed with the project group until a consensus was reached.

### Development of Design Solutions

An initial interaction design was developed that described the functions and processes in the interaction between the system and user. For the development, requirements identified by the analysis of the context of use and a literature research as well as international recommendations for navigation and user dialogue design [11-13] were taken into account. In addition, the results of a competitor analysis and existing paper-based checklists served as sources of information. The medical content was based on a checklist provided by the German Cognitive Aid Working Group for the intraoperative emergency of malignant hyperthermia. On the basis of the initial interaction design, the first prototype was built in *Balsamiq Mockups 3* (Balsamiq Studios, LLC) [14]. Subsequently, a demonstration video was created, describing the individual screens of the prototype regarding their functionality and navigation options, and was made available online.

A revised interaction design was developed, considering the results of the evaluation of the first prototype and the specifications of the main requirements document. On the basis of the revised interaction design, the second prototype was built using *PowerPoint*. The development was realized by the creation of a separate slide for each view and each function call within the application. Furthermore, an audio track was recorded for every slide, describing the respective screen regarding the functionality and navigation options. Subsequently, 3 separate demonstration videos were produced (for desktop/tablet general structure, desktop/tablet exemplary run with checklists, and smartphone general structure), which were made available online.

The third prototype was created for tablets. The process was identical to the second prototype concerning the graphical generation. This time, however, most of the elements were hyperlinked to other slides, allowing logical navigation between the different screens of the prototype. As a result, once the PDF had been opened with a PDF reader, the user could interactively navigate the prototype.

### Evaluation of Design Solutions

#### Web-Based Questionnaire

To evaluate the first and second prototype, corresponding Web-based questionnaires were developed. Except for the open questions, the items were answered on a 5-point Likert scale, ranging from 1=strongly disagree to 3=neither agree nor disagree (neutral) to 5=strongly agree. For some items, respondents were asked to justify their response should the rating be neutral or negative (ie, rating 1 to 3). The evaluation of the first prototype included the System Usability Scale (SUS) [15,16]. The final questionnaires (adapted from the German versions) can be found in Multimedia Appendix 1.

For evaluation purposes, the developed questionnaires were provided via the platform *SoSci Survey* [17], including a link to the respective demonstration videos. The links to the SoSci Survey portal were distributed to the German Cognitive Aid Working Group via email. Project partners were encouraged to forward the links to interested colleagues to increase the number of respondents. For the analysis, the average rating and the SDs were calculated for all closed questions. The answers to open questions were grouped by topic, and the number of participants mentioning the topic was counted. The identified issues to improve the cognitive aid were prioritized to indicate the most urgent areas for revision. The prioritization was based on the frequency of mention, the severity of the problem, and the requirements to better adapt the application to the medical workflow without overloading it. The results of the evaluations were summarized in tables and text form, and aspects for revision of the application were listed by thematic classification.

#### Think-Aloud Protocol and Paper Questionnaire

The third prototype was evaluated by means of a laboratory-based think-aloud protocol as a well-established method to capture all actions and thoughts when using a system [18-20]. The think-aloud study was led by a researcher of the Chair of Medical Informatics and conducted at a single institution (Department of Anesthesiology, University Hospital Erlangen). The department’s library provided an undisturbed and quiet environment for the study. The related institutional review board approved the investigation. Participants were end users who were neither familiar with the project nor with the interface design. The individual meetings were scheduled to last for about 20 to 30 min.

All participants were introduced to a medical emergency (malignant hyperthermia) and asked to imagine themselves being in the situation. In addition, the test persons were confronted with 4 tasks that had to be solved using the prototype. After all test conditions were explained, participants were asked to work on the tasks in the given order. At a test session, all verbal statements and screen actions were videotaped via the device.

After completing the think-aloud protocol, a test person was asked to complete a paper questionnaire related to the previous use of the prototype. The questionnaire included the SUS. Following the evaluation, the recorded videos were transcribed and the results were thematically summarized and compiled together with the evaluation of the questionnaires in tabular form.

The test instructions and tasks for the think-aloud protocol as well as the questionnaire (adapted from the German versions) can be found in Multimedia Appendix 2.

## Results

### Analysis of the Context of Use

**Figure 2:**
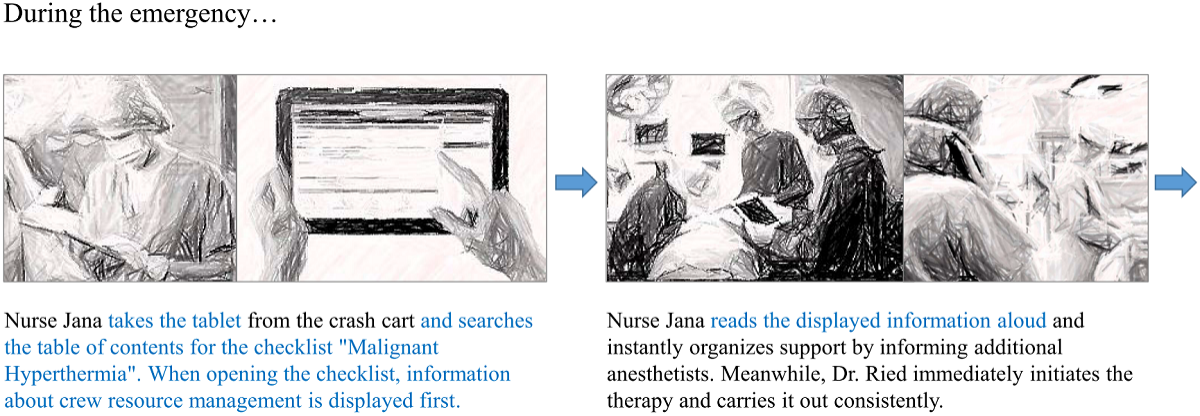
Scenario of the cognitive aid (use case malignant hyperthermia, use of the application via a tablet device).

Anesthesiologists in the inpatient and outpatient area were identified as the primary target group for the digital cognitive aid. Anesthesia nurses as part of the anesthesia team are considered secondary users. Figure 2 shows an excerpt of the storyboard for emergency malignant hyperthermia (adapted from the German version), which describes the use of the cognitive aid on a tablet device. In the presented scenario, an anesthesia nurse takes on the role of a reader.

### Specification of the User Requirements

The main requirements document, including the results of the user context analysis and evaluation of the first prototype, has 52 pages and is available in German only. The document can be requested from the corresponding author on reasonable demand.

**Figure 3:**
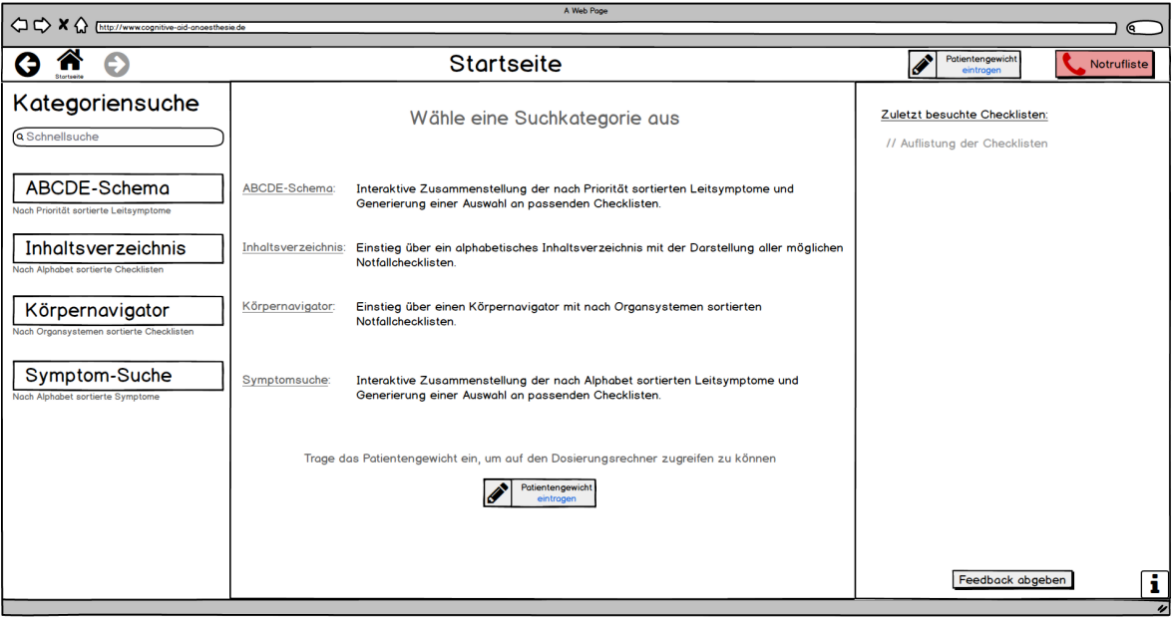
First prototype start page (only available in German).

### Development of Design Solutions

#### First Prototype

On the basis of the initial interaction design, the first prototype of the digital cognitive aid was created as individual screenshots of a desktop application. At the time of the creation, colors were deliberately used very sparingly, as in this first step the focus was on functionality and screen layout of the cognitive aid. Figure 3 illustrates the start page of the application. From here, the respective emergency information can be found via the different search options offered on the left (keyword search, search by the airway, breathing, circulation, disability, exposure (ABCDE) approach, alphabetical search, search via body navigator, and search for symptoms). Once a checklist is opened, this area can be used to navigate through various sections of the emergency information. The central and largest area of the 3-column page structure always displays the essential information. Here, the user receives a short description of the various available functions. This is also the section where the search results are displayed, and after opening a checklist, the corresponding emergency action steps are displayed. The content of the right-hand area is variable: on the start page, the area has a chronological function and displays the recently accessed checklists. Once a checklist is called up, additional information about the emergency action steps is displayed at this position. In the bar at the top right of the screen, the patient’s weight can be entered for individual drug dosage suggestions and an emergency call list can be accessed.

#### Second Prototype

The second prototype was built as a desktop or tablet and smartphone version. The prototypes were intentionally kept as similar as possible and differed only in parts of the navigation and screen layout. The revised interaction design included the following major changes compared with the initial interaction design: 2-column layout for the desktop or tablet version, selection of patient type (infant, adult, or parturient) before selecting a search option, 4 offered search options (keyword or free-text search, alphabetical search, ABCDE approach search, or body navigator search), possibility to check off completed action steps, illustration of checklist content by different tabs, and display of the elapsed time since opening a checklist.

#### Third Prototype

**Figure 4:**
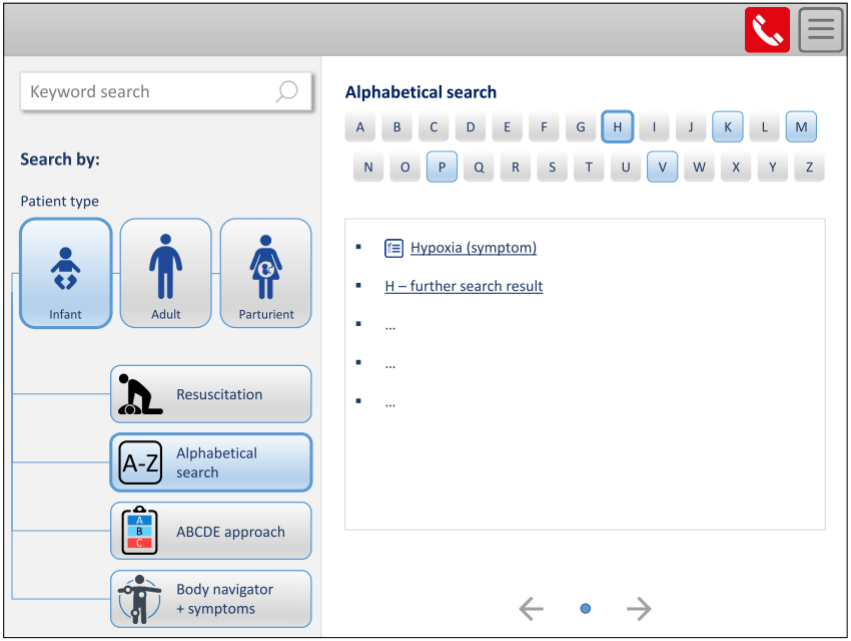
Third prototype, patient type infant selected, alphabetical search (adapted from the German version).

The third prototype was modified in accordance to the revised and supplemented requirements resulting from the evaluation of the second prototype. Figure 4 illustrates the start page of the third prototype with the opened alphabetical search. In the left area, different search options to find emergency checklists can be selected, and except for the keyword search, the patient type has to be chosen first. On the right, a corresponding letter can be marked, to which the respective search results are listed in the area below.

**Figure 5:**
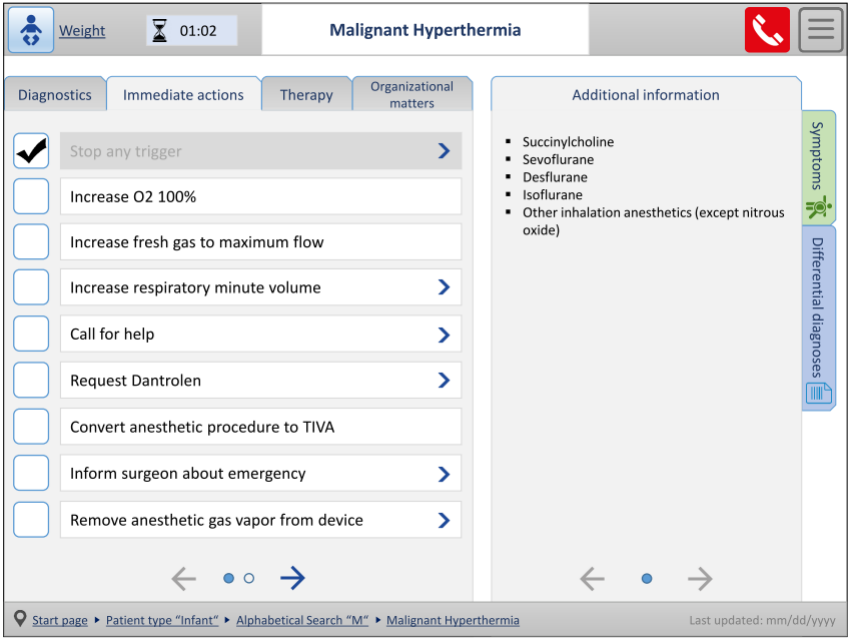
Third prototype, checklists view malignant hyperthermia (adapted from the German version).

An example of how a checklist (malignant hyperthermia) is displayed in the cognitive aid prototype is shown in Figure 5. The checklist title is centered at the top, and the time elapsed since the checklist was opened is positioned to the left, together with the selected patient type and optional patient weight. To the right, an emergency call list and the menu icon are depicted. In the middle of the screen, the emergency action steps are displayed on the left side, with different sections being selectable via the tabs above. Completed action steps can be marked as performed by ticking off. Additional information on individual items is provided in the right-hand area. Furthermore, related symptoms and differential diagnoses can be accessed via tabs on the right. In the bottom bar, the navigation path to the checklist search and information concerning the last update of the checklist content are displayed.

Additional screenshots of the third prototype (adapted from the German version) can be found in Multimedia Appendix 3.

### Evaluation of Design Solutions

#### First Prototype: Web-Based Questionnaire

Sample description: A total of 12 anesthesiologists participated in the Web-based survey, whose affiliation was either the Hannover Medical School, the Hospital Munich, the University Hospital Erlangen, the Trauma Hospital Berlin, or not specified. The participant structure consisted of 5 senior physicians, 6 resident physicians, and 1 specialist, with an average professional experience of 12 years (range: 1 to 25 years). Half of the participants had already gained experience with paper checklists, but no comparable systems or applications had been used so far. The general computer literacy level was rated medium to high.

The evaluation of the SUS scores resulted in an average value of 74 (SD 12), indicating good usability of the prototype. Thereby, 2 participants rated the application less than 60 (considerable usability problems), 6 participants between 60 and 80 (borderline to good usability), and 4 participants over 80 (good to excellent usability).

The analysis of the questionnaire resulted in a prioritized list of identified topics and requirements to be clarified. After discussion with the project group, the following final list of modifications and additional requirements emerged:

- The additional information should continue to be listed in the right-hand area.
- No scrolling should be offered, as otherwise the user could lose track or miss important points.
- A combination search of symptoms is not desired as the application is not intended to be a decision support system.
- The ABCDE approach and symptom search provide redundant information. As a result, the symptom search should be completely removed.
- The body navigator should not allow the simultaneous selection of multiple organ systems.
- An additional subdivision of the search into the patient categories *Infant*, *Adult*, and *Parturient*, intended as a preliminary query right after the start of the application, should be included. After selecting one of these categories, an automatic query of the patient’s weight should appear, which is only optional.
- On the start page, one of the search categories should be displayed in the middle area instead of a short explanation of the individual functions.

**Table 1:** Average time required per task during the think-aloud protocol.

Task	Time (seconds), mean (SD)
Find the checklist for malignant hyperthermia using the alphabetical search and open it	19 (11)
Mark the first 3 emergency action steps as completed	6 (4)
Navigate back to the start page and use the body navigator to find other emergencies or symptoms that can cause circulatory problems	44 (8)
Open the malignant hyperthermia checklist and inform yourself about possible differential diagnoses via the respective tab	20 (6)

#### Second Prototype: Web-Based Questionnaire

Sample description: A total of 8 anesthesiologists participated in the Web-based survey, whose affiliation was either the Hannover Medical School, the University Hospital Erlangen, the University Hospital Heidelberg, or not specified. The participant structure consisted of 2 senior physicians, 4 resident physicians, and 2 specialists, with an average professional experience of 10 years (range: 1 to 25 years). On average, respondents rated their computer literacy level as medium (advanced user). Furthermore, 2 of the participants already worked with paper-based checklists and 5 with electronic checklists (eg, apps or own checklists of the university hospital). In case of an emergency, the intranet (n=3) or colleagues (n=2) were mainly used for further information. The desktop computers (n=7) or smartphones (n=6) are mainly used to search for information in everyday life. Furthermore, the participants preferred to use a digital cognitive aid for the desktop computer (n=6) and smartphone (n=6).

The page layout, design, search options, positioning of the elements, and the way the information is presented were predominantly positively evaluated for both prototype variants (desktop or tablet and smartphone). Especially, the uniform design of the different variants was appreciated, as it allowed easy orientation and comparable handling across different devices. The presentation of an open checklist was perceived to be helpful, as it resembled an open book with indexes.

The revised requirements that resulted from a joint discussion in the project group on the results of the questionnaire were, among others, the following:

- Add quick selection for cardiopulmonary resuscitation checklists to the search options.
- When selecting an organ system in the body navigator, the associated symptoms as well as the corresponding checklists should both be listed.
- Crew resource management (CRM) and literature tab should only be available via the menu button.
- The size of the top bar and the tabs should be slightly increased.
- Tabs and times should additionally be marked with pictograms.
- A stopwatch should only be displayed when one of the resuscitation emergencies is selected.

#### Third Prototype: Think-Aloud Protocol and Paper Questionnaire

Sample description: A total of 9 anesthesiologists participated in the think-aloud protocol and completed the questionnaire. The participants had an average professional experience of 8 years (range: 1 to 20 years). All respondents rated their computer literacy level as medium. Furthermore, 6 of the participants already worked with paper-based checklists and 4 with electronic checklists. The majority stated that they were familiar with the use of a tablet in everyday life (mean 3.33 [SD 1.56]). It was almost a unanimous agreement among participants that they could well imagine using a tablet in the operating room (mean 4.44 [SD 0.96]).

The evaluation of the SUS scores resulted in an average value of 77 (SD 15). One participant rated the application less than 60, 4 participants between 60 and 80, 4 participants over 80, and 1 of them 95 (almost perfect application).

Overall, the application was rated positively by the participants regarding the concept (n=2), design (n=3), function (n=2), intuitiveness (n=5), and structure (n=3). Furthermore, it was easy to understand after a short practice (n=3). Participants noticed that the button for start page navigation was too small (n=4), a home button was missing (n=3), the areas of the body navigator were confusing (n=4), training was necessary (n=2), and too many clicks and tabs existed (n=2).

The participants needed an average of 88 seconds (SD 19) to complete all tasks set for the think-aloud protocol. The results of the individual tasks are listed in Table 1.

## Discussion

### Principal Findings

#### Discussion of Results

This study addresses our research questions on the clinicians’ demands on the digital representation of a cognitive aid for emergency situations and how its user interface can be best developed that meets user requirements, is highly user-friendly, and is easy to navigate.

Within a UCD process, requirements were identified and refined step by step, resulting in a user-centered development of a digital cognitive aid for intraoperative crisis management in anesthesia.

Storyboards were developed to create a common vision for developers and end users about the context of use and enabled the specification of the first user requirements. The content for the storyboards was based on initial requirements defined in a brainstorming session with the project group and context interviews that identified the characteristics of future users as well as the peculiarities of the anesthetic workplace. In addition, a competition analysis and literature search contributed to the development of a first interaction design, facilitating the creation of a first prototype. A user evaluation of the first prototype helped to define and prioritize further requirements. Considering these new requirements, a revised interaction design was developed. On the basis of this design, a second prototype was created, which in turn was evaluated in a user test. Subsequently, this process was repeated with a third prototype.

The evaluation of the SUS scores of the first and third prototype proved that the iterative process described above improved usability. Furthermore, the number of requirements to be revised was reduced from step to step. The evaluation of the third user test also revealed that the application was highly intuitive even without previous instruction. The search options allowed the user to find a checklist in a relatively short time and to check off the action steps quickly and easily. In addition, some of the test persons indicated the feeling of being familiar with the application after a short training period. This test result provides a solid basis for a subsequent test of the application under real-life conditions, associated with the high stress of an emergency situation. For this purpose, the project plan stipulates the evaluation of the cognitive aid during simulated scenarios of an intraoperative crisis. This evaluation may identify further requirements for the application that could not be determined with the previous evaluation methods.

The development of the cognitive aid needs to focus primarily on the main user of the application, which in the context of the German health care system would be the physician anesthesiologist. In contrast, anesthesia nurses as part of the anesthesia team play an assisting role and are considered as secondary users. However, the usage requirements identified were largely identical for both user groups. Major differences between both user groups only exist with regard to the handling of the cognitive aid. As the use of a digital cognitive aid for the management of intraoperative crises is not yet established in Germany, the question is yet unresolved whether anesthesia nurses will be given the task of taking the team through the checklist (ie, as readers) or whether they will continue to merely carry out the required treatment steps. In a simulation study, the presence of a reader improved the performance in crisis situations and the completeness of task execution [21].

At present, knowledge concerning the actual use of cognitive aids for crisis management in anesthesia is limited. Chances are that only a few anesthesia departments have implemented the use of paper-based checklists in their crisis management approach. Available data are mainly derived from simulation studies [5]. Although the use of the World Health Organization Surgical Safety Checklist is mandatory in Germany [22], this type of checklist only refers to the perioperative standardization of procedures. However, for the treatment of intraoperative emergencies, there exists no such mandatory regulation. Emergency checklists are therefore only available as a voluntary hospital-based measure for patient safety.

This study was able to identify the following main requirements for a digital cognitive aid: intuitive operation and error tolerance of the design, short navigation paths, full functionality for offline use, and the provision of an educational concept for those anesthesia departments that decide to implement the cognitive aid. These results are confirmed by data from other studies. In the manual by Crosland et al for the development of digital emergency checklists, the aspects of error tolerance and navigation are already provided with short implementation conditions [23]. Marshall et al concluded that the roles for the use of a cognitive aid must be clearly defined to help all team members to perform their tasks in a coordinated order [24]. The implementation of a cognitive aid should always be preceded by appropriate introductory training: on the one hand, because users can experience the physical limits of the application in the real environment and, on the other hand, because familiarity with the application increases both effectiveness and frequency of use. To overcome the concern that an application might lose its functionality in case of internet connection failure [25], it is recommended to ensure functional independence from the internet [26].

There are not many recent publications on digital cognitive aids for crisis management in anesthesia. However, it is striking that the majority of digital emergency checklists found on this topic relate mainly to the field of resuscitation. One of these publications is the tablet-based *Decision Support Tool* application for the management of advanced life support activities developed by McEvoy et al [27]. The functionality is comparable with that of the developed third prototype. Thus, the application also features a 2-column layout and includes an initial selection of the patient type and to check off action steps. In comparison, the application mainly differs in the navigation concept, which is far less clear and is associated with poorer use of the screen space. Information about the development process or the evaluation of user satisfaction is not available. An innovative concept of the cognitive aid described in this paper is that it aims to cover all emergencies, non-normal situations, and symptoms encountered during an intraoperative crisis in the future. Along with non-normal events, the list is expected to include 80 to 100 topics. In addition, an editor functionality is considered, which would enable the user to tailor the checklists to local conditions (eg, trade names of available drugs, depositories of critical drugs, location of the nearest defibrillator, and emergency telephone numbers).

Another point that distinguishes this paper from the publications mentioned is that none of the applications address issues of CRM, such as provision of role clarity, distribution of work load, identification of all available resources, reevaluation of the situation, and clear communication. This finding was also confirmed by Evans et al, who conducted an evaluation of the content and usability of the checklists and complained about the lack of explicit statements on responsibility [28]. The requirement analysis for the cognitive aid confirmed the initial idea that the integration of team management aspects was indeed a desirable feature. Although this requirement was already considered in the first prototype (as a placeholder), the development of a concrete concept for the implementation of CRM support in the application turned out to be a major difficulty. This challenge became apparent in the different development stages of the prototypes. The first version included a switch for activating or deactivating CRM prompts implemented as pop-ups. A separate tab was provided for CRM in the second prototype, which could be used to read relevant notes. In the third version, CRM was no longer available as a switch or button, but it was intended to integrate the relevant information directly into the action points. As the elaboration of medical contents is a task in itself and is therefore not a part of this study, the elaboration of CRM support will not be further discussed. It should be noted, however, that a regulated distribution of tasks is essential for effective emergency management to be supported by a cognitive aid.

#### Discussion of Methods

The prototypical development of the digital cognitive aid for anesthesia to support intraoperative crisis management was based on the UCD process [7] described by the following main subactivities: analysis of the context of use, specification of usage requirements, development of design solutions and evaluation from the user’s perspective, and extension of usage requirements. With the UCD, the end users play a key role in the development process right from the beginning, enabling optimal adaptation of the application to their specific needs.

The lack of usability can lead to rejection of the system or, even worse, to treatment errors that jeopardize patient safety [29]. The working context of the cognitive aid may further aggravate usability problems as a result of the cognitive limitations that occur during an intraoperative emergency.

The majority of diagnosis and treatment errors in anesthesia occur as a result of a mismatch between situational requirements and the actor’s mental model of the current situation (ie, the so-called *human error*) [30]. Fortunately, human performance in time-critical situations can be enhanced by the use of medical applications that have been developed with user-oriented methods [31].

Finally, a user-oriented development process is a prerequisite for approval as a medical device [32]. Development according to the UCD process is therefore an established and thus suitable method for the conception of a digital cognitive aid for crisis management in anesthesia.

The usability of a software product always depends on the real operating conditions, making an analysis of the working context an indispensable prerequisite [7]. Herczeg recommends a combination of empirical and analytical methods for a comprehensive analysis [33]. In this study, the characteristics and requirements of the user groups and the user environment were analyzed by means of several different methods. The knowledge gained helped to understand anesthesiologists’ working practices and needs as well as the complexity of the anesthesia workplace in routine and emergency situations.

By user tests of the different prototypes during the iterative UCD process, each development step was evaluated regarding its usability. Thus, the application was checked with each step for suitability to the intended purpose and adapted accordingly. This enables a high user acceptance of a future application.

### Limitations

Throughout the entire development process, participants were only anesthesiologists from hospitals but not from outpatient departments. As a result, it is possible that the requirements for intraoperative emergencies in the outpatient and inpatient settings only partially overlap.

In addition, only physicians as the primary user group participated in evaluations. The requirements of nurses may therefore differ from those identified.

Furthermore, all evaluations of the prototypes took place in theory, that is, without dealing with a real emergency situation. However, these disadvantages are counterbalanced by future plans to evaluate the cognitive aid during performance of an anesthesia team in a simulation study.

### Conclusions

Anesthesiology—as an acute medical field—is particularly characterized by its high demands on decision making and action in uncertain, dynamic, or time-critical situations. The fact that emergency checklists for anesthesia have so far only been available as paper versions has prompted the BDA to develop a national digital cognitive aid for crisis management in anesthesia.

Applications intended for use in stressful and time-critical situations pose special demands on their development. The integration of usability aspects is essential for everyday and emergency suitability. For this reason, a user-centered development was chosen according to the UCD process model as defined in ISO 9241-210 [7].

The UCD process allowed us to develop a prototypical digital cognitive aid, exhibiting high usability and user satisfaction in the demanding environment of anesthesiological emergencies. Both aspects are essential to increase the acceptance of the application in later stages.

The presented study approach, combining many different methods for determining user requirements, may be useful for other implementation projects in a highly demanding environment.

## Appendix

Multimedia Appendix 1 Questionnaires first and second prototype.

Multimedia Appendix 2 Think-aloud protocol and questionnaire third prototype.

Multimedia Appendix 3 Screenshots third prototype.

## Abbreviations

ABCDE: airway, breathing, circulation, disability, exposure
BDA: Professional Association of German Anesthesiologists
CRM: crew resource management
ISO: International Organization for Standardization
SUS: System Usability Scale
UCD: user-centered design
