# Supplementary material for: A Digital Cognitive Aid for Anesthesia to Support Intraoperative Crisis Management: Results of the User-Centered Design Process
Source: JMIR Mhealth Uhealth. 2019 Apr 29;7(4):e13226. doi: 10.2196/13226 (PMC6658227; doi:10.2196/13226)
Supplement: Multimedia Appendix 1 [file mhealth_v7i4e13226_app1.pdf]

Cognitive Aid: Questionnaire for the user tests of the first and second prototype

List of questions

With the exception of the open questions, the following questions were answered using a five-point Likert scale. The individual gradations were:

- 1 = Strongly disagree
- 2 = Disagree
- 3 = Neither agree nor disagree
- 4 = Agree
- 5 = Strongly agree

First Prototype

System Usability Scale (10)

- I think that I would like to use this system frequently.
- I found the system unnecessarily complex.
- I thought the system was easy to use.
- I think that I would need the support of a technical person to be able to use this system.
- I found the various functions in this system were well integrated.
- I thought there was too much inconsistency in this system.
- I would imagine that most people would learn to use this system very quickly.
- I found the system very cumbersome to use.
- I felt very confident using the system.
- I needed to learn a lot of things before I could get going with this system.

Design (4)

- I consider the presentation of the information in a three-column layout to be clearly arranged.
- Steps that have already been completed or chapters that have been visited should be visually highlighted or marked.
- Instead of the presentation in an extra column, additional information should be integrated in the text (e.g. by expanding arrows).
- Symptoms of differential diagnoses should be able to be checked so that the verification does not have to be started over and over again with each new differential diagnosis.

Navigation (4)

- I feel the navigation is simple and self-explanatory.
- The application allows an easy and quick change between the different areas (the different entry options or checklist contents).
- I find the display of the possible navigation direction by navigation arrows clear and understandable.
- Instead of the possibility to "browse" through the contents, I would have wished for a possibility to "scroll".

<u>Features</u> (21)

ABCDE approach

- I feel the structure and presentation of the ABCDE approach to be logical/clear.
    - If result = Neither agree nor disagree/ disagree/ strongly disagree:
      What exactly interferes with the structure or display of the ABCDE approach?
- The representation of all selected symptoms in the right area makes sense to me.
- The filtering function when searching for "AND" (combination) or "OR" (intersection) makes sense to me.

Table of contents

- I feel the structure and presentation of the table of contents to be logical/clear.
    - If result = Neither agree nor disagree/ disagree/ strongly disagree:
      What exactly do you find disturbing about the structure or the presentation of the table of contents?
- It is helpful to be able to view information on the selected checklist in advance (in the right-hand area) without having to call up/open the checklist.

Body navigator

- I feel the structure and the representation of the body navigator to be logical/clear
    - If result = Neither agree nor disagree/ disagree/ strongly disagree:
      What exactly do you find disturbing about the structure or the representation of the Body Navigator?
- I would have preferred several organ systems to be selectable at the same time.

Symptom search

- I feel the structure and presentation of the symptom search to be logical/clear
    - If result = Neither agree nor disagree/ disagree/ strongly disagree:
      What exactly do you find disturbing about the structure or display of the symptom search?
- The filtering function when searching for "AND" (combination) or "OR" (intersection) makes sense to me.

Emergency call

- I think it is good that the emergency numbers are displayed in a pop-up window.
- The positioning of the emergency call function is well chosen.

Enter patient weight

- I think it is good that an extra window ("pop-up window") is opened to enter the patient's weight.
- I would have preferred the patient weight to be queried automatically when starting the application.
- When querying the patient weight, I would have preferred an input option in "grams" (instead of "kilograms" as shown).

Dosage calculator

- The dosage calculator contains all the functions I need in my daily work routine.
  - If result = Neither agree nor disagree/ disagree/ strongly disagree:
    Which features were missing in the dosage calculator?
- I like that the dosage calculator is displayed in an additional column (right screen area).
  - If result = Neither agree nor disagree/ disagree/ strongly disagree:
    What would be your alternative presentation recommendation for the dosage calculator?

Reminder function

- I think the possibility to activate action reminders by clicking the "double arrow" icon before action steps is well chosen.
- The positioning of the reminder function in the third column (right screen area) is well selected.
  - If result = Neither agree nor disagree/ disagree/ strongly disagree:
    What would be your alternative presentation recommendation for the reminder display?

General

- The application contains all the functions I need for effective and efficient emergency management.
  - If result = Neither agree nor disagree/ disagree/ strongly disagree:
    Which function do you think is missing?

Open questions

- What are the three things you liked best about the prototype so far?
- What would be the three things that need most revision to make the application better?

Sociodemographic questions (6)

- How many years of professional experience do you have?
- From which clinic/facility do you come (free text)?
- Which professional group do you belong to? (Chief Physician/Head Physician/Assistant Physician/Other)
- How do you generally assess your computer skills/knowledge? (high: I am very experienced and technically talented; mediocre: I get along well with most systems; low: I find many systems difficult to use.)
- Have you ever used paper-based checklists or Cognitive Aids for emergency situations? (no; yes, the following paper checklists/CAs)
- Have you ever used similar applications ("checklist apps")? (no; yes, the following applications)

Second Prototype

Part 1: Evaluation of the desktop/tablet version

Start page of the application and the various search options (9)

- The start page is clearly structured.
- The various search options are easy for the user to recognize.
- The start page is designed in such a way that a combination of patient type and alphabetical search/search via the ABCDE approach or search via the body navigator is easily possible.
- The symbols/icons used (telephone list; further options: imprint, feedback, patient type) are clear and easy to understand.
- In addition to the search options already offered, I would have liked to have an additional search option for the checklist category (Reanimation/ACLS, Emergencies, Non-Normal Situations, Symptoms).
- In my opinion too many input steps (clicks) have to be made to find a checklist.
- I am satisfied with the display of the alphabetical search (selection of letters, display of associated and linked checklists).
    - If result = Neither agree nor disagree/ disagree/ strongly disagree:
      In your opinion, what would be a better design option for the alphabetical search?
- I am satisfied with the display of the search via the ABCDE approach (checkable symptoms structured according to ABCDE and display of associated/linked checklists).
    - If result = Neither agree nor disagree/ disagree/ strongly disagree:
      In your opinion, what would be a better design option for the ABCDE approach?
- I am satisfied with the display of the search via the body navigator (graphical selection of the organ system via pictograms and display of related/linked checklists).
    - If result = Neither agree nor disagree/ disagree/ strongly disagree:
      In your opinion, what would be a better design option for the body navigator?

Organization and structure of a checklist page (13)

- The page of a checklist is clearly structured.
- The organization/division of a checklist page is easy to understand.
- I am satisfied with the basic division of the checklist contents into two screen areas.
- I am satisfied with the display of the checklist sections diagnostics, immediate measures, therapy, organization (variable, depending on checklist) or A/B/C/D/E (variable, depending on checklist) on the left side of the screen.
- I am satisfied with the display of additional information, CRM measures, symptoms, differential diagnoses, literature on the right side of the screen.
- I would have liked symptoms and differential diagnoses to also be displayed on the left side of the screen (e.g. as a tab next to the immediate measures).
- I think it's good to be able to check off the action steps.
- I wish I could check off the symptoms as well.
- I wish I could check off the differential diagnoses as well.
- I think it's good that the patient's weight is only queried when opening the selected checklist and not when searching for a checklist.

- I am satisfied with the positioning of the following information in the upper screen area:
    - Patient type
    - Weight
    - Time elapsed since opening the checklist
    - Checklist name
    - Timer (stopwatch)

    - If result = Neither agree nor disagree/ disagree/ strongly disagree:
      What alternative positioning would you suggest? Please enter the information type (e.g. patient type) and your proposal here:
- I am satisfied with the positioning of the following information in the lower screen area:
    - Navigation path
    - Display of the actuality of the checklist
    - PDF retrieval possibility of the complete checklist incl. additional information

    - If result = Neither agree nor disagree/ disagree/ strongly disagree:
      What alternative positioning would you suggest? Please enter the information type (e.g. navigation path) and your proposal here:
- I am satisfied with the weight input option.
    - If result = Neither agree nor disagree/ disagree/ strongly disagree:
      What alternative design would you suggest?

<u>Reminder of CRM actions and open action steps</u> (5)

- The reminder/alarm is designed in such a way that it does not distract or irritate the user and does not interfere with his task performance.
- The warning symbols used (red warning triangle, red exclamation mark) are suitable for attracting the immediate attention of the user.
- The user can understand the meaning of the warning symbols.
- In addition to displaying warning symbols, I would have liked a pop-up window to appear (and thus interrupt) with the respective reminder (CRM reminder, reminder for open action steps).
- I am satisfied with the positioning of the warning symbols.
    - If result = Neither agree nor disagree/ disagree/ strongly disagree:
      What alternative positioning would you suggest?

<u>Task support</u> (12)

- There are aspects of the application that are difficult to understand.
    - If result = Neither agree nor disagree/ disagree/ strongly disagree:
      What exactly is difficult for you to understand?
- The implemented functionality meets my expectations. All important and expected functions have been implemented.
    - If result = Neither agree nor disagree/ disagree/ strongly disagree:
      Which functions do you think are missing?
- The execution of a function always leads to the expected result.
    - If result = Neither agree nor disagree/ disagree/ strongly disagree:
      Where not?

- All required information can be found quickly in the application.
    - If result = Neither agree nor disagree/ disagree/ strongly disagree: Which information is more difficult to find?
- Errors during input (e.g. accidental clicking of action steps, accidental selection of an option, selection of the wrong patient group) can be easily undone.
- I find the correction effort for errors to be low.
- It would be possible for me to "process" a checklist in a reasonable time with the help of the application.
- All checklist information is presented in such a way that you always have an overview (e.g. what has been done, what still needs to be done, where you are in the checklist).
- I can complete my work steps in the order that makes the most sense to me.
- The application is self-explanatory and intuitive to use.
- The application is easy to learn without outside help or a manual.
- I think, even with rare use it is no problem to find your way back into the application.

Navigation (2)

- The application offers me good handling possibilities to move within the checklist (tabs, buttons, paging arrows, additional information, hyperlinks).
- I find the possibility to navigate within and between parts of a checklist uniform.

Layout and design in general (8)

- The individual pages are well designed.
    - If result = Neither agree nor disagree/ disagree/ strongly disagree: Which page/s especially not?
- The design of the application is visually appealing.
- The representation of the information has a sufficiently good contrast.
- The colors are sufficiently discreet.
- Overall, I am satisfied with the current prototype for desktop/tablet.
- (Open question) What do you like most about the desktop/tablet prototype?
- (Open question) What do you think needs the most improvement and why?
- (Open question) Do you have a proposal for a name for the application or a concise abbreviation? (The used name and logo are to be understood only as placeholders.)

Part 2: Evaluation of the smartphone version

Start page of the application and the various search options (2)

- The start page is clearly structured.
- The various search options are easy for the user to recognize.

Organization and structure of a checklist page (5)

- The page of a checklist is clearly structured.
- The organization/division of a checklist page is easy to understand.
- The icons/icons used (phone list; additional options: Imprint, feedback, CRM, symptoms, differential diagnoses etc.) are clear and easy to understand.
    - If result = Neither agree nor disagree/ disagree/ strongly disagree: Which symbol(s) specifically not?

- I am satisfied with the positioning of the following information in the upper screen area:
    - Patient type
    - Weight
    - Time elapsed since opening the checklist
    - Checklist name
    - Timer (stopwatch)

    - If result = Neither agree nor disagree/ disagree/ strongly disagree:
      What alternative positioning would you suggest? Please enter the information type (e.g. patient type) and your proposal here:
- I am satisfied with the positioning of the following information in the lower screen area:
    - Navigation path
    - Display of the actuality of the checklist
    - PDF retrieval possibility of the complete checklist incl. additional information

    - If result = Neither agree nor disagree/ disagree/ strongly disagree:
      What alternative positioning would you suggest? Please enter the information type (e.g. navigation path) and your proposal here:

Task support (8)

- All required information can be found quickly in the application.
    - If result = Neither agree nor disagree/ disagree/ strongly disagree:
      Which information is more difficult to find?
- It would be possible for me to "process" a checklist in a reasonable time with the help of the application.
- All checklist information is presented in such a way that you always have an overview (e.g. what has been done, what still needs to be done, where you are in the checklist).
- I can complete my work steps in the order that makes the most sense to me.
- The application is self-explanatory and intuitive to use.
- The application is easy to learn without outside help or a manual.
- I think, even with rare use it is no problem to find your way back into the application.
- In the smartphone version I could do without the following functions for reasons of clarity:
    - Time elapsed since opening the checklist
    - Timer (stopwatch)
    - Reminders

Navigation (2)

- The application offers me good handling possibilities to move within the checklist (tabs, buttons, paging arrows, additional information, hyperlinks).
- I find the possibility to navigate within and between parts of a checklist uniform.

Layout and design in general (7)

- The individual pages are well designed.
  - If result = Neither agree nor disagree/ disagree/ strongly disagree: Which page/s especially not?
- The design of the application is visually appealing.
- The representation of the information has a sufficiently good contrast.
- The colors are sufficiently discreet.
- Overall, I am satisfied with the current prototype for smartphone.
- (Open question) What do you like most about the smartphone prototype?
- (Open question) What do you think needs the most improvement and why?

Part 3: Personal data

Sociodemographic questions (10)

- What institution are you from? (please specify)
- Your gender? (female/male)
- How long do you work in your current field of expertise? (in years)
- What is your current position? Resident, Specialist, Senior Physician, Others (please specify)
- If you were to assess your computer skills, what would you call yourself? (beginner (1), advanced (2), expert (3))
- Are you currently working with paper-based checklists? If so, which ones?
- Are you currently working with electronic checklists? If so, which ones?
- What sources of information do you use in an emergency? Internet, Intranet, costume-bag textbooks, colleagues, others (please specify)
- If you are looking for information in everyday life, which devices do you mainly use (multiple answers possible)? (smartphone/tablet/desktop)
- For which of the following devices would you prefer to use the application? (smartphone, tablet, desktop)

Individual notes (1)

- The last part of the questionnaire is reserved for your individual comments. There is room here for further criticism of the application or for problems that you have not been able to get rid of when answering the questions:
