# Supplementary material for: A Digital Cognitive Aid for Anesthesia to Support Intraoperative Crisis Management: Results of the User-Centered Design Process
Source: JMIR Mhealth Uhealth. 2019 Apr 29;7(4):e13226. doi: 10.2196/13226 (PMC6658227; doi:10.2196/13226)
Supplement: Multimedia Appendix 2 [file mhealth_v7i4e13226_app2.pdf]

Cognitive Aid: User test of the third prototype

Test instruction think-aloud protocol

You will receive tasks that you should try to solve with the help of the prototype. It is important that you say loudly what is going on in your head. Just say what you perceive and what you would do next. For example, you could say something like:

- - "The start page makes an overloaded impression on me."
- - "The feedback from the app has made me insecure!"
- - "...now I'm thinking about how..."
- - "The meaning of the buttons is a mystery to me."

You are welcome to ask questions to the study director during the test, but they may not be answered directly.

Situation:

- You are in an emergency situation in the operating room.
- Your patient is a 6-year-old child.
- The child shows the following symptoms: rise in temperature, tachycardia, trismus
- Your working diagnosis is malignant hyperthermia (MH).
- An electronic checklist on a tablet is now available to support the emergency situation.

Task 1:

- Find the appropriate checklist using the alphabetical search function
- Select the checklist

Task 2:

The Malignant Hyperthermia (MH) checklist is open. You have read and performed the first 3 of the listed immediate measures.

- Mark the first 3 emergency action steps as completed (tick off)

Task 3:

In addition to malignant hyperthermia (MH), other emergencies can also cause circulatory problems.

- Navigate back to the start page
- Use the body navigator to find other emergencies or symptoms that may cause circulatory problems

Task 4:

You are uncertain about your working diagnosis.

- Open the malignant hyperthermia (MH) checklist
- Inform yourself about possible differential diagnoses via the corresponding tab (end of the test)

Questionnaire on the third prototype

With the exception of the open and sociodemographic questions, the following questions were answered using a five-point Likert scale. The individual gradations were:

- 1 = Strongly disagree
- 2 = Disagree
- 3 = Neither agree nor disagree
- 4 = Agree
- 5 = Strongly agree

System Usability Scale (10)

- I think that I would like to use this application frequently.
- I found the application unnecessarily complex.
- I thought the application was easy to use.
- I think that I would need the support of a technical person to be able to use this application.
- I found the various functions in this application were well integrated.
- I thought there was too much inconsistency in this application.
- I would imagine that most people would learn to use this application very quickly.
- I found the application very cumbersome to use.
- I felt very confident using the application.
- I needed to learn a lot of things before I could get going with this application.

Tablet use (2)

- I am used to using a tablet in everyday life.
- I could well imagine using a tablet in the operating room.

General findings (3)

- (Open question) What I noticed positively was this:
- (Open question) What I noticed negatively was this:
- (Open question) In addition, I noticed the following:

Sociodemographic questions (6)

- How do you rate your computer skills in general? (high, I am very experienced and technically gifted; mediocre, I get along well with most systems; low, I find many systems to operate)
- I have already used an emergency checklist during a clinical emergency (posters, index cards, book, etc.) (yes/no)
- I've used a digital emergency checklist before during a clinical emergency (yes/no)
- Gender (female/male)
- Profession (nurse/physician)
- Years of work experience in your profession (in years)
