# Supplementary material for: A Digital Cognitive Aid for Anesthesia to Support Intraoperative Crisis Management: Results of the User-Centered Design Process
Source: JMIR Mhealth Uhealth. 2019 Apr 29;7(4):e13226. doi: 10.2196/13226 (PMC6658227; doi:10.2196/13226)
Supplement: Multimedia Appendix 3 [file mhealth_v7i4e13226_app3.pdf]

Multimedia Appendix 3 – Screenshots third prototype

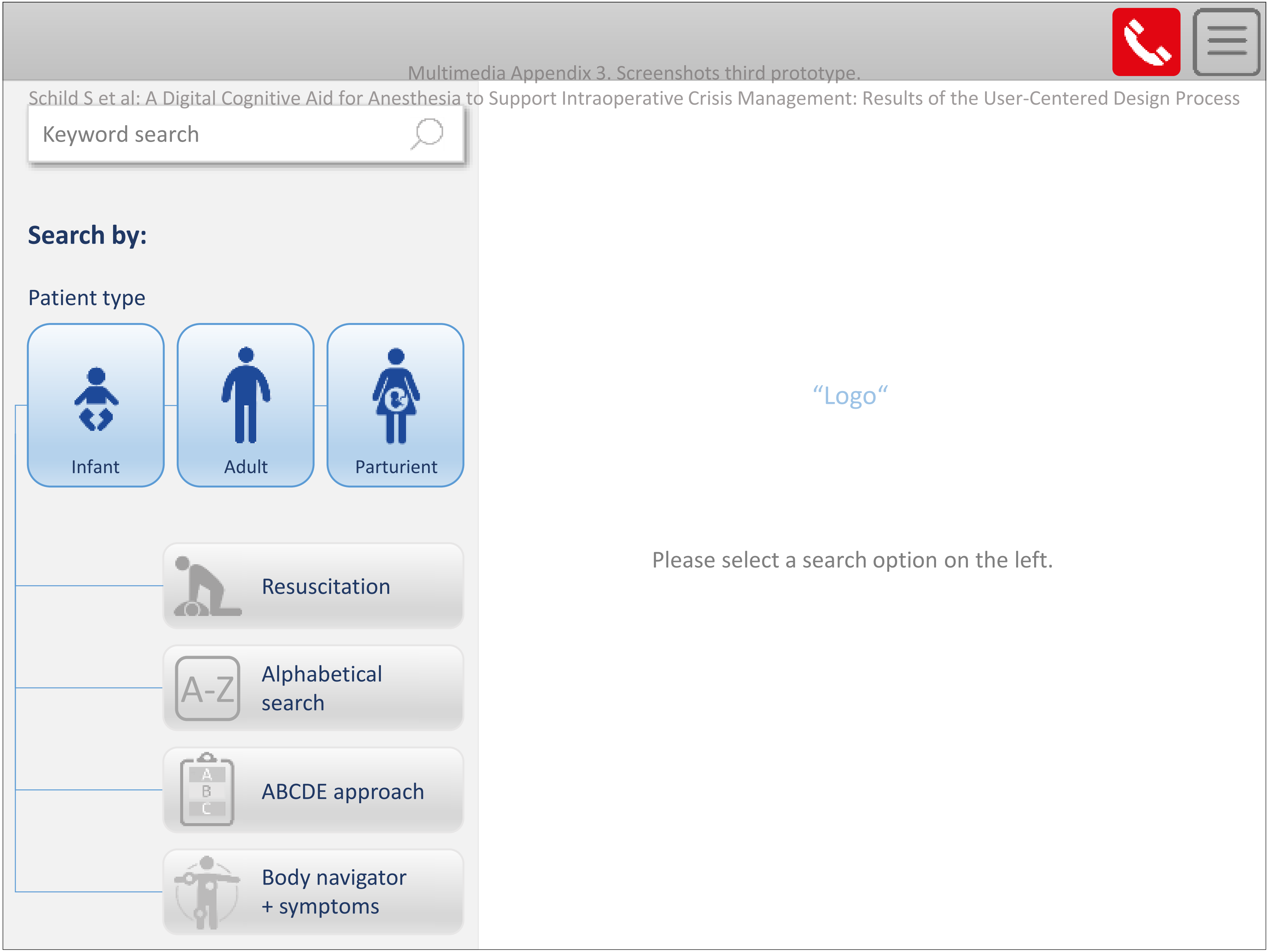
Keyword search
Search by:
Patient type
Infant
Adult
Parturient
Resuscitation
A-Z
Alphabetical search
A
B
C
ABCDE approach
Body navigator + symptoms
"Logo"
Please select a search option on the left.

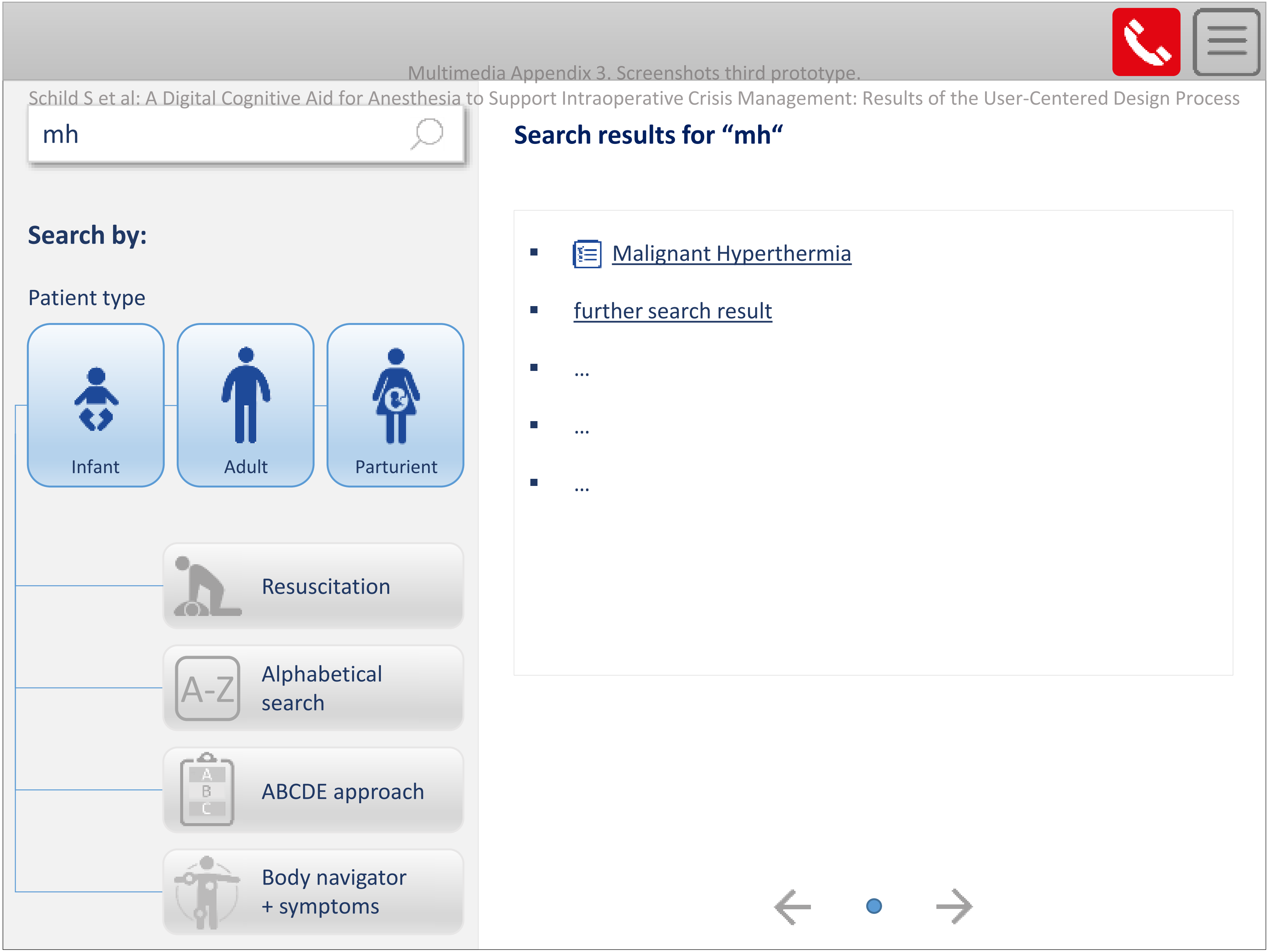
mh
Search by:
Patient type
Infant
Adult
Parturient
Resuscitation
Alphabetical search
A-Z
ABCDE approach
A
B
C
Body navigator + symptoms
Search results for “mh“
Malignant Hyperthermia
further search result
…
…
…

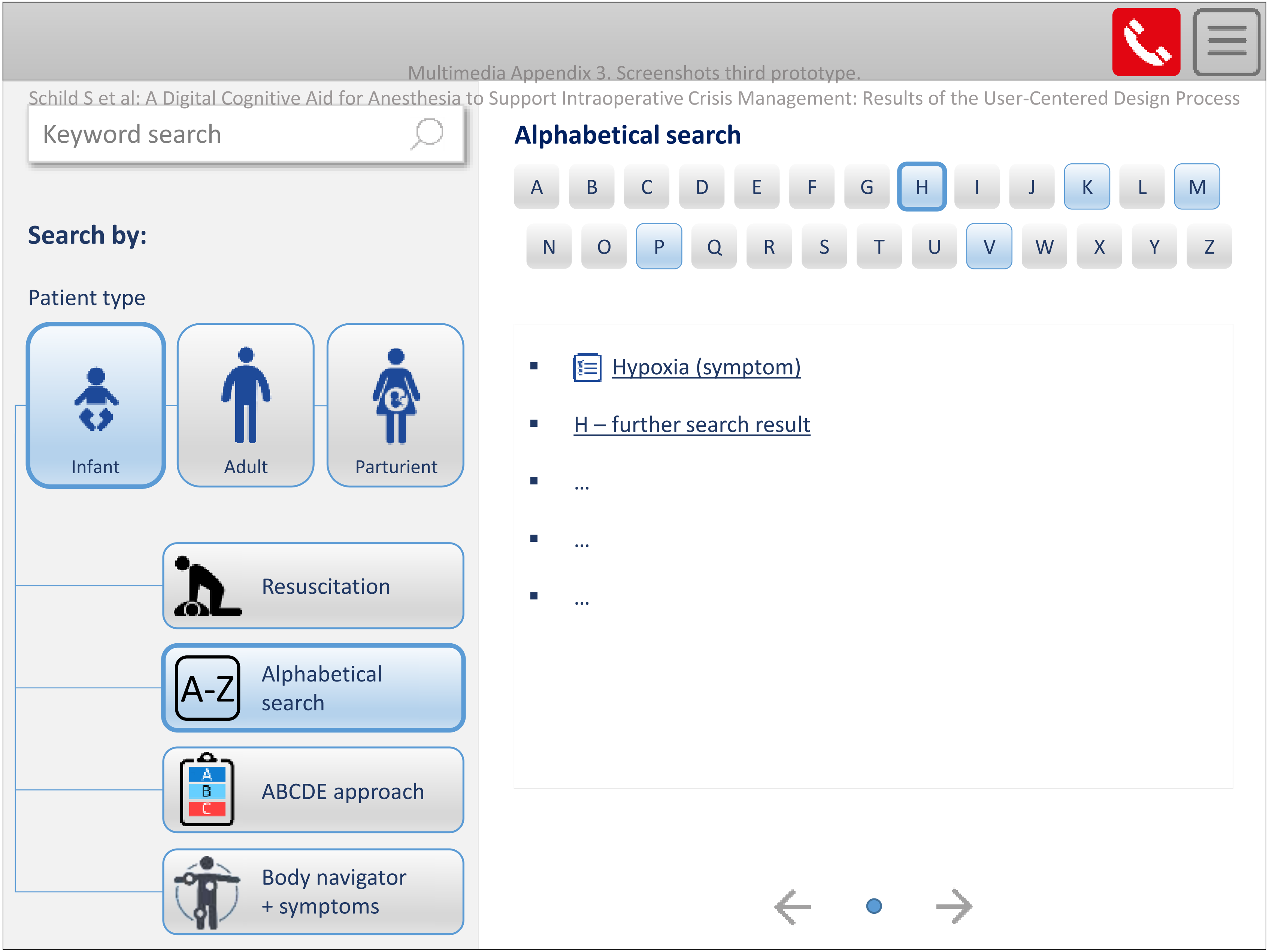
Keyword search
Alphabetical search
A B C D E F G H I J K L M
N O P Q R S T U V W X Y Z
Search by:
Patient type
Infant
Adult
Parturient
Hypoxia (symptom)
H – further search result
…
…
…
Resuscitation
A-Z
Alphabetical search
A
B
C
ABCDE approach
Body navigator + symptoms

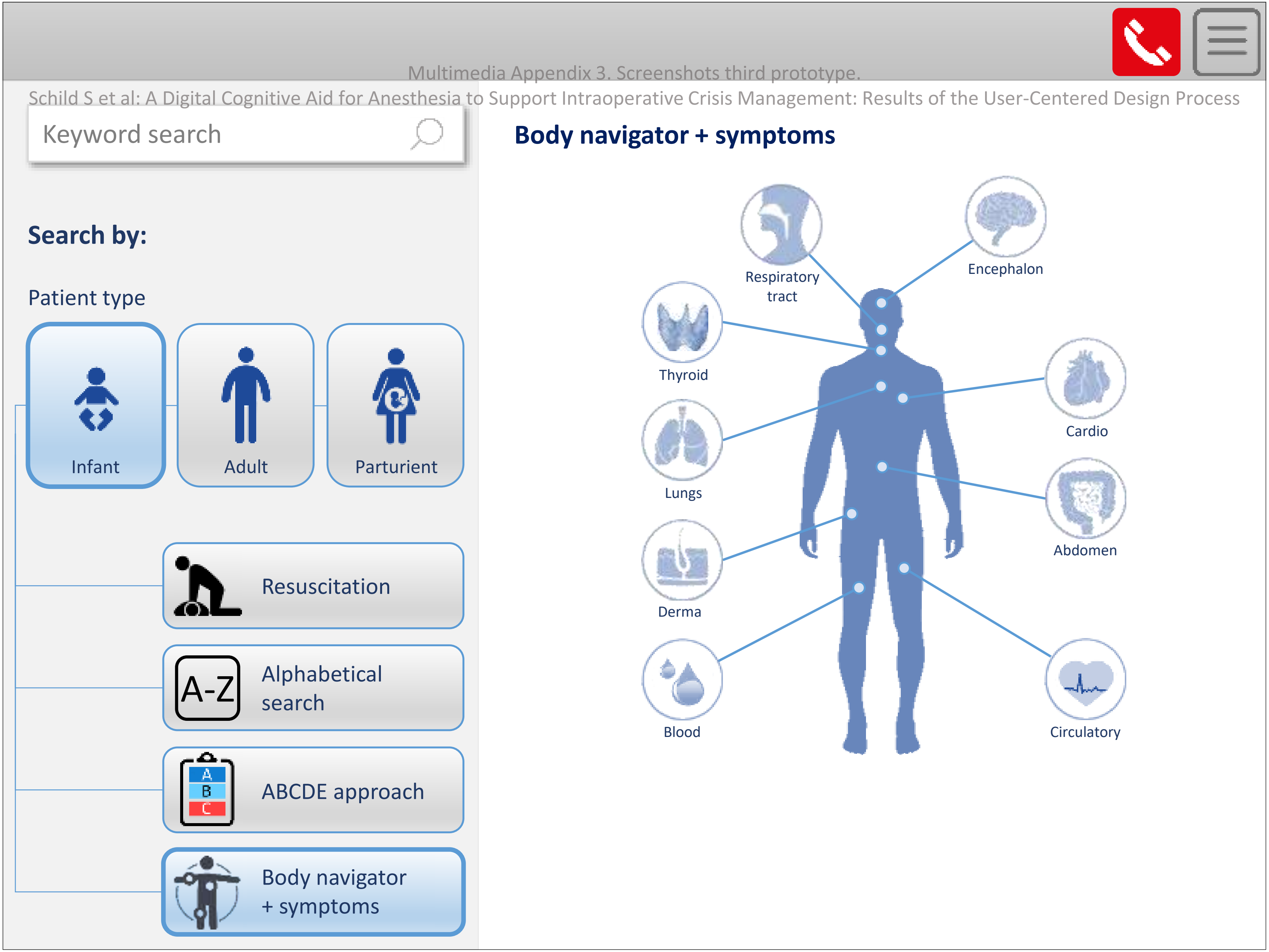
Keyword search
Body navigator + symptoms
Search by:
Patient type
Infant
Adult
Parturient
Resuscitation
A-Z
Alphabetical search
A
B
C
ABCDE approach
Body navigator + symptoms
Respiratory tract
Encephalon
Thyroid
Cardio
Lungs
Abdomen
Derma
Blood
Circulatory

Malignant Hyperthermia

Diagnostics | Immediate actions | Therapy | Organizational matters | Additional information

- Stop any trigger
- Increase O_2 100%
- Increase fresh gas to maximum flow
- Increase respiratory minute volume
- Call for help
- Request Dantrolen
- Convert anesthetic procedure to TIVA
- Inform surgeon about emergency
- Remove anesthetic gas vapor from device

Symptoms during anesthesia induction

- Masseter spasm following succinylcholine
- Generalized muscle rigor

Symptoms during anesthesia maintenance

- Acute increase in $EtCO_2$
- Hypoxia
- Increased oxygen consumption
- Tachykardia
- Mixed metabolic-respiratory acidosis
- Profuse sweating
- Marmorated skin
- Hyperthermia
- Rhabdomyolysis
- Acute kidney injury
- Disseminated intravascular coagulation
- Bleeding tendency

Symptoms | Differential diagnoses

Weight

01:02

Malignant Hyperthermia

Diagnostics | Immediate actions | Therapy | Organizational matters

- [ ] Stop any trigger >
- [ ] Increase O2 100%
- [ ] Increase fresh gas to maximum flow
- [ ] Increase respiratory minute volume >
- [ ] Call for help >
- [ ] Request Dantrolen >
- [ ] Convert anesthetic procedure to TIVA
- [ ] Inform surgeon about emergency >
- [ ] Remove anesthetic gas vapor from device >

Additional information

- Succinylcholine
- Sevoflurane
- Desflurane
- Isoflurane
- Other inhalation anesthetics (except nitrous oxide)

Symptoms

Differential diagnoses

Start page ▸ Patient type "Infant" ▸ Alphabetical Search "M" ▸ Malignant Hyperthermia

Last updated: mm/dd/yyyy

Malignant Hyperthermia

Multimedia Appendix 3. Screenshots third prototype.

Schild S et al: A Digital Cognitive Aid for Anesthesia to Support Intraoperative Crisis Management: Results of the User-Centered Design Process

Diagnostics | Immediate actions | Therapy | Organizational matters

- [x] Stop any trigger
- [ ] Increase O2 100%
- [ ] Increase fresh gas to maximum flow
- [ ] Increase respiratory minute volume
- [ ] Call for help
- [ ] Request Dantrolen
- [ ] Convert anesthetic procedure to TIVA
- [ ] Inform surgeon about emergency
- [ ] Remove anesthetic gas vapor from device

Additional information

- Succinylcholine
- Sevoflurane
- Desflurane
- Isoflurane
- Other inhalation anesthetics (except nitrous oxide)

Symptoms

Differential diagnoses

Weight 01:02

Malignant Hyperthermia

Multimedia Appendix 3. Screenshots third prototype.

Diagnostics | Immediate actions | Therapy | Organizational matters

- [x] Stop any trigger
- [x] Increase O2 100%
- [ ] Increase fresh gas to maximum flow
- [ ] Increase respiratory minute volume
- [ ] Call for help
- [ ] Request Dantrolen
- [ ] Convert anesthetic procedure to TIVA
- [ ] Inform surgeon about emergency
- [ ] Remove anesthetic gas vapor from device

Additional information

Symptoms

Differential diagnoses
